# Understanding the Crucial Role of Phosphate and Iron Availability in Regulating Root Nodule Symbiosis

**DOI:** 10.1093/pcp/pcae128

**Published:** 2024-10-26

**Authors:** Mariel C Isidra-Arellano, Oswaldo Valdés-López

**Affiliations:** Department of Trait Diversity and Function, Royal Botanic Gardens, Kew, Richmond TW9 3AE, UK; Laboratorio de Genómica Funcional de Leguminosas, Department of Biology, Facultad de Estudios Superiores Iztacala, Universidad Nacional Autónoma de México, Tlalnepantla 54090, México

**Keywords:** Nutrient homeostasis, Phosphate and iron deficiency, Phosphate starvation response system, Root nodule symbiosis

## Abstract

The symbiosis between legumes and nitrogen-fixing bacteria (rhizobia) is instrumental in sustaining the nitrogen cycle and providing fixed nitrogen to the food chain. Both partners must maintain an efficient nutrient exchange to ensure a successful symbiosis. This mini-review highlights the intricate phosphate and iron uptake and homeostasis processes taking place in legumes during their interactions with rhizobia. The coordination of transport and homeostasis of these nutrients in host plants and rhizobia ensures an efficient nitrogen fixation process and nutrient use. We discuss the genetic machinery controlling the uptake and homeostasis of these nutrients in the absence of rhizobia and under symbiotic conditions with this soil bacterium. We also highlight the genetic impact of the availability of phosphate and iron to coordinate the activation of the genetic programs that allow legumes to engage in symbiosis with rhizobia. Finally, we discuss how the transcription factor phosphate starvation response might be a crucial genetic element to integrate the plant’s needs of nitrogen, iron and phosphate while interacting with rhizobia. Understanding the coordination of the iron and phosphate uptake and homeostasis can lead us to better harness the ecological benefits of the legume-rhizobia symbiosis, even under adverse environmental conditions.

## Introduction

Nitrogen is a crucial building block for diverse biomolecules, including nucleic acids and proteins. Although nitrogen is abundant in the atmosphere, most living organisms cannot directly metabolize it. Instead, they can only metabolize fixed forms (e.g. ammonium and amino acids), and plants can metabolize other forms of nitrogen, notably nitrate (Zhong et al. 2023). However, nitrate is scarce in the soil, negatively impacting plant growth and development.

Nitrogen fertilizers are widely used to increase crop yield, but overuse harms soil and water bodies. Root endosymbiosis is a sustainable strategy that can help to reduce our dependence on synthetic fertilizers (Singh et al. 2023). For instance, legumes engage in symbiosis with nitrogen-fixing soil bacteria collectively known as rhizobia. Legumes host rhizobia into specialized root organs called nodules, inside which rhizobia fix the atmospheric nitrogen (Roy et al. 2020). Legumes obtain fixed nitrogen through this symbiosis, significantly enhancing their growth in nitrogen-deficient soils and serving as a natural biofertilizer (Zahran 1999). In exchange, rhizobia obtain carbohydrates as a carbon source and diverse mineral nutrients, including phosphorus (P) and iron (Fe) (Roy et al. 2020).

The first step in establishing a successful symbiosis between legumes and rhizobia begins with the roots recognizing rhizobia. Once rhizobia are recognized, diverse molecular events are activated in the host legume, which are required for a successful symbiosis (Roy et al. 2020). For instance, rapid and sustained oscillation of nuclear and perinuclear calcium concentrations, known as calcium spiking, is a crucial molecular event that activates the genetic machinery, enabling legumes to host rhizobia (Roy et al. 2020). Decoding these calcium signatures activates the transcription of a cohort of transcription factor (TF)–encoding genes, including *Nodule Inception* (*NIN*) (Schauster et al. 1999). NIN controls different stages of root nodule symbiosis, from rhizobial infection to nodule organogenesis (Schauster et al. 1999, Marsh et al. 2007, Breakspear et al. 2014, Liu et al. 2019, Feng et al. 2021).

Legumes growing symbiotically require more Fe and P than plants providing adequate added nitrogen fertilizer. It is estimated that up to 20–30% of total plant P and Fe is allocated toward nodules (Sulieman and Tran 2015, González-Guerrero et al. 2023). This high demand for Fe and P is vital to ensure optimal nitrogen-fixing activity (Kouas et al. 2005, González-Guerrero et al. 2014). Fe and P play diverse roles in plant growth and root nodule symbiosis. For instance, Fe is a cofactor of leghemoglobin, an oxygen-carrying protein required for symbiotic nitrogen fixation and one of the essential cofactors required to assemble the nitrogenase complex that catalyzes nitrogen fixation (González-Guerrero et al. 2023). A subset of P’s roles includes the decoding of the nod factor (NF) signal and synthesizing ATP to fix atmospheric nitrogen symbiotically (Sulieman and Tran 2015). Similar to other nutrients required for root nodule symbiosis (e.g. calcium), the host legumes must tightly regulate the homeostasis of these two nutrients to ensure the necessary amount for its development and to sustain the symbiotic nitrogen fixation process. Defects in the plant P/Fe uptake and homeostasis, as well as deficiency of these mineral nutrients in the soil, compromise the establishment and functioning of the root nodule symbiosis. Indeed, P deficiency significantly reduces nodule formation in diverse legumes (Hernández et al. 2009, Cabeza et al. 2014, Isidra-Arellano et al. 2020). Fe or P deficiency substantially reduces the nitrogen fixation rate (Hernández et al. 2009, González-Guerrero et al. 2023). Because of their relevance in the establishment and function of the root nodule symbiosis, significant efforts to understand the genetic mechanisms underlying the homeostasis, transport and translocation of Fe and P in legumes while interacting with rhizobia have been made. By understanding how legumes sustain Fe and P homeostasis during the root nodule symbiosis, we will be better positioned to leverage the ecological benefits of this symbiosis and develop sustainable agriculture. Here, we review and discuss current knowledge about how Fe and P plant levels modulate the establishment and functioning of root nodule symbiosis. We also discuss how the host legume might genetically regulate the interplay between Fe, P and nitrogen.

## Establishment of the Root Nodule Symbiosis

The symbiosis between legumes and rhizobia is established under nitrogen-deficient conditions. Under such nutritional conditions, the host plant excretes flavonoid compounds to the rhizosphere, where compatible rhizobia detect them. In turn, rhizobia produce lipo-chitooligosaccharides known as NFs (Roy et al. 2020). Legumes perceive NFs through receptor-like kinases, including nod factor receptor 5 (NFR5) and NFR1 in *Lotus japonicus* and their orthologous nod factor perception (NFP) and lysine motif kinase 3 (LYK3) in *Medicago truncatula* (Amor et al. 2003, Madsen et al. 2003, Radutoiu et al. 2003). NFR5/NFP and NFR1/LYK3 form a protein complex to recognize NFs, which is mediated by the rhizobia infection receptor–like kinase 1 (RinK1) in *L. japonicus* (Zhou et al. 2024). NFs’ perception changes the stability and localization of NFR5/NFP and NFR1/LYK3 at the plasma membrane by compartmentalizing them into nanodomains at the root hair tips (Zhou et al. 2024).

NFR5/NFP and NFR1/LYK3 transduce the symbiotic signal to the malectin-like/leucine-rich repeat receptor kinase, symbiosis receptor–like kinase (SYMRK), in *L. japonicus* or its counterpart does not make infections 2 (DMI2) in *M. truncatula* (Endre et al. 2002, Stracke et al. 2002). Four serine residues at the alpha-I motif of SYMRK/DMI2 are phosphorylated, which creates a docking site required to propagate the rhizobial signal (Abel et al. 2024). Upon SYMRK/DMI2 activation, this protein kinase propagates the symbiotic signal to the nucleus of plant root cells through the interactions and phosphorylation of diverse proteins (Roy et al. 2020). For instance, Early Phosphorylated Protein 1 (EPP1) is a potential interactor of SYMRK/DMI2 required to activate calcium spiking in *M. truncatula* (Ferrer-Orgaz et al. 2024). Indeed, the overexpression of a phospho-mimic version of *EPP1* partially rescues the symbiotic phenotype of SYMRK/DMI2 in *M. truncatula*, demonstrating that this genetic component is instrumental in perpetuating the rhizobial signal from the plasma membrane to the nucleus (Ferrer-Orgaz et al. 2024).

The protein kinase Ca^2+^/calmodulin-dependent protein kinase (CCaMK) or its counterpart does not make infections 3 (DMI3) decodes these calcium signatures in *L. japonicus* and *M. truncatula*, respectively (Roy et al. 2020). CCaMK/DMI3 phosphorylates the TF CYCLOPS/IPD3, subsequently activating the master TF *NIN* (Singh and Parniske 2012, Singh et al. 2014). NIN promotes intracellular rhizobial infection and nodule organogenesis and controls the nodule number (Soyano et al. 2014, Roy et al. 2020). NIN also controls the maturation of the nodule to the nitrogen fixation state (Feng et al. 2021). This transition to functional nodules is mediated by proteolytic processing by the DNF1 complex (Feng et al. 2021). This DNF1-dependent proteolytic process results in a carboxyl-terminal NIN fragment containing the DNA-binding domain, which activates a cohort of genes associated with symbiosome development and nitrogen fixation in both model legumes *M. truncatula* and *L. japonicus* (Feng et al. 2021).

Tremendous advances in understanding how the legume host transduces the rhizobial signal have been made in the past 20 years. However, knowledge gaps need to be improved on our understanding of the genetic control of the root nodule symbiosis. For instance, we still need to fully understand how the symbiotic signal is perpetuated from the plasma membrane to the nuclei or how the nutritional status of the host plant modulates the decoding of the rhizobial signal. This knowledge will allow us to better harness the ecological benefits of this symbiosis even under adverse environmental conditions. Moreover, filling these gaps of understanding will also contribute to achieving the ambitious goal of transferring the symbiotic nitrogen fixation trait to other non-leguminous plants.

## Genetic Control of Nodule Formation

Diverse phytohormones, including small-secreted peptide hormones, participate in nodule formation (Taleski et al. 2018, Velandia et al. 2022). For instance, C-terminally encoded peptides (CEPs), apart from controlling nitrogen-demand signaling and lateral root development, also promote nodule formation in *M. truncatula* (Imin et al. 2013, Taleski et al. 2018). *MtCEP1* is induced under low-nitrogen conditions (Imin et al. 2013). MtCEP1 peptide is transported via the xylem from the root to the shoot, where it is detected by the compact root architecture 2 (CRA2) receptor (Hault et al. 2014, Mohd-Radzman et al. 2016). The perception of MtCEP1 by CRA2 triggers the synthesis of the microRNA miR2111, which is transported to the root to target the *Kelch-repeat-containing F-box protein too much love* (*TML*), a negative regulator of nodule formation (Nishimura et al. 2002, Takahara et al. 2013, Tsikou et al. 2018, Gautrat et al. 2020). The degradation of *TML* is required for legumes to be developmentally competent for nodulation (Imin et al. 2013). Indeed, applying synthetic MtCEP1 peptide or its overexpression increases the number of nodules in *M. truncatula* (Imin et al. 2013). Another CEP required to promote nodule formation is MtCEP7 (Laffont et al. 2020, Ivanovici et al. 2023). Unlike MtCEP1, *MtCEP7* is upregulated in response to rhizobia or purified NF, and its expression is regulated by NIN (Laffont et al. 2020, Ivanovici et al. 2023). Hence, legumes have co-opted mechanisms related to nitrogen perception and signaling to regulate nodule formation and grow on soils with low mineral nitrogen (reviewed in Taleski et al. 2018).

Obtaining fixed nitrogen through the symbiosis with rhizobia is energetically costly for the host plant. Therefore, legumes tightly regulate the number of nodules to avoid an energetic imbalance and compromise their growth and development. Legumes have evolved an intricate long-distance signaling pathway called autoregulation of nodulation (AON) to restrict the number of nodules (Ferguson et al. 2019). This pathway is initiated by producing the CLE-related-root signal 1 (CLE-RS1) and CLE-RS2 peptides in *L. japonicus* or their counterparts MtCLE12 and MtCLE13 in *M. truncatula* (Mortier et al. 2010, Ferguson et al. 2019). The production of these CLE peptides begins with the first rhizobia-induced cortical cell division and continues through nodule development and symbiotic nitrogen fixation (Ferguson et al. 2019). NIN regulates the transcriptional activation of these CLE peptide–encoding genes in *L. japonicus* (Soyano et al. 2014). The mature CLE peptides are transported via the xylem from the root to the shoot, where they are perceived by the receptor hypernodulation and aberrant root formation 1 (HAR1) in *L. japonicus* or super numeric nodules (SUNN) in *M. truncatula* (Nishimura et al. 2002, Ferguson et al. 2019). The CLE peptide perception activates a signaling pathway that produces shoot-derived signals, which are transported to the root to inhibit further nodule development (Ferguson et al. 2019).

Available or excess fixed nitrogen suppresses root nodule symbiosis, and legumes employ a non-symbiotic mechanism to uptake nitrogen from the soil. Legumes have also evolved a pathway to inhibit nodulation when fixed nitrogen (e.g. nitrate) is available. In this pathway, at least two NIN-like proteins (NLPs), NLP1 and NLP4, regulate the expression of nitrate-responsive genes (Lin et al. 2018, Nishida et al. 2020). Under optimal nitrogen conditions, NLP1 and NLP4 translocate to the nucleus and interact with NIN, thereby repressing the expression of critical genes (i.e. *CRE1* and *NF-YA1*) for nodule formation, resulting in inhibition of the root nodule symbiosis (Lin et al. 2018, Nishida et al. 2018). Therefore, legumes have developed signaling pathways to manage the trade-off between benefits and costs associated with root nodule symbiosis. Part of these signaling pathways might operate under environmental conditions in which the energetic cost of this endosymbiosis compromises the legume development. This hypothesis has been studied in diverse legumes under nutrient-deficient conditions. Recent data suggest that legumes use part of these pathways to modulate the root nodule symbiosis according to the plant host’s nutritional status (Isidra-Arellano et al. 2020, Ito et al. 2024).

## Phosphate and Fe Homeostasis in a Non-Symbiotic Scenario

P is a vital macronutrient for living organisms, including plants. It is fundamental for signal transduction via protein phosphorylation and is a critical component in the biosynthesis of ATP and membrane lipids during cell growth and division. Indeed, P is instrumental in sustaining the membrane lipid biosynthesis during nodule development and for rhizobia in differentiating bacteroids (Zhang et al. 2020). Although total P is abundant in soil, the availability of phosphate (Pi), the preferred form of P uptake by plants, often constrains plant growth and development because of its high chemical fixation and slow diffusion in soil (Vance et al. 2003).

**Fig. 1:**
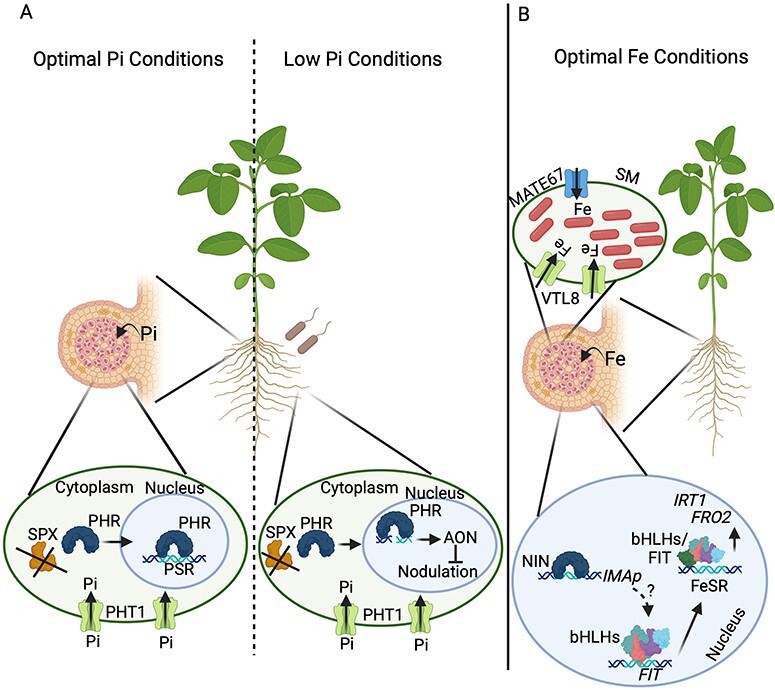
Genetic mechanisms controlling the Pi and Fe transport in root nodules. (A) Under optimal Pi conditions, legumes activate the TF PHR in both rhizobia-infected and non-infected nodule cells by suppressing the interaction with SPX proteins. Once PHR is active, this TF is translocated to the nucleus, which activates the expression of genes confirming the PSR system, including those encoding *phosphate high affinity transporters 1* (PHT1). When legumes grow under Pi-limiting conditions, PHR activates the expression of genes of the AON pathway, which limits the interaction with rhizobia, thereby reducing the formation of nodules. (B) Under optimal Fe conditions, the TF NIN activates the expression of *iron man peptides* (*IMAp*). Then, IMAp might promote that bHLH TFs activate the expression of *FIT*. Then, FIT interacts with bHLH TFs to activate the Fe starvation response (FeSR) system, which includes *iron transporter 1* (*IRT1*) and *FRO2*. Once the FeSR system is active, Fe must be transported from the plant-infected cells to the bacteroid. To do so, diverse Fe transporters, including MATE67 and VTL8, located at the SM deliver, the Fe required for bacteroids to fix the atmospheric nitrogen. Images were created using BioRender (https://biorender.com).

Plants have developed diverse strategies to obtain Pi and secure its efficient use (Zhang et al. 2014). These functions include membrane lipid remodeling, metabolic adjustment, Pi uptake, translocation and homeostasis (Zhang et al. 2014). Genes participating in these responses conform to the Pi starvation response (PSR) system (Isidra-Arellano et al. 2021). PSR genes are transcriptionally regulated by the TF phosphate starvation response (PHR). PHR’s activity is post-translationally regulated according to the plant Pi status (Isidra-Arellano et al. 2021). Under Pi-sufficient conditions, inositol pyrophosphate, a sensor of cell Pi status, binds to SPX proteins, promoting their interaction with PHR in *Arabidopsis thaliana* and rice (Isidra-Arellano et al. 2021). The interaction with SPX proteins prevents the PHR translocation to the nucleus, inhibiting the PSR system’s activation in *A. thaliana* and rice (**Fig. 1A**) (Isidra-Arellano et al. 2021). PHR is also phosphorylated by the GSK3/SHAGGY-Like Kinase2 (GSK2) under Pi-sufficient conditions in rice (Zhang et al. 2024). This phosphorylation at serine residue 269 suppresses the DNA-binding activity of Os-PHR2 (Zhang et al. 2024). These two levels of regulation inhibit the activation of the PSR system to avoid Pi overaccumulation under optimal Pi conditions. Under Pi-limiting conditions, SPX proteins are degraded, and the GSK2–PHR interaction is no longer promoted (Zhang et al. 2024). Once PHR is active, this TF activates the expression of genes encoding for *high-affinity H^+^/Pi transporters of the Phosphate transporter 1* (*PHT1*) family as well as Pi exporters of the *PHOSPHATE 1* (*PHO1*) family (Isidra-Arellano et al. 2021). PHT1 and PHO1 allow Pi uptake and translocation in diverse plant species, including leguminous plants (**Fig. 1A**) (Isidra-Arellano et al. 2021). The activity of PHT1 and PHO1 is post-translationally regulated by the E2 ubiquitin conjugase PHOSPHATE 2 (PHO2) and the E3 ubiquitin ligase Nitrogen Limitation Adaptation 1 (NLA1) in *A. thaliana* (Bari et al. 2006, Liu et al. 2012, Lin et al. 2013). In addition, when cellular Pi levels are low, the microRNAs *miR399* and *miR827* accumulate and target the *PHO2* and *NLA* mRNA, allowing PHT1 cotransporters and PHO1 to be localized at the plasma membrane in *A. thaliana* (Bari et al. 2006, Liu et al. 2012, Lin et al. 2013). Once plants satisfy their Pi needs, the long non-coding RNA (LncRNAs) *Induced by Phosphate Starvation 1* (*IPS1*) is accumulated in *A. thaliana* (Franco-Zorrilla et al. 2007). Similarly, the LncRNAs *Mt4*, homolog of *IPS1*, and the *Deficiency-Induced LncRNA 1* (*PDIL1*) are accumulated in *M. truncatula* (Wang et al. 2017). *IPS1*/*Mt4* and PDIL*1* sequester miR399, thereby protecting *PHO2* from miR399-mediated cleavage. Moreover, *PDIL2* and *PDIL3* directly target Pi transporter encoding genes belonging to the PHT family in *M. truncatula* (Wang et al. 2017). Hence, plants use diverse mechanisms to maintain the Pi homeostasis accordingly (Franco-Zorrilla et al. 2007, Wang et al. 2017).

Fe is a fundamental micronutrient required for various metabolic processes, including photosynthesis, respiration, chlorophyll biosynthesis and nitrogen fixation (González-Guerrero et al. 2023). Fe is abundant on Earth, but unlike P, Fe is highly reactive to oxygen, and the formation of insoluble oxidized Fe restricts its uptake by roots, especially in high-pH and calcareous soils. Fe also interacts with Pi, forming insoluble complexes, making it inaccessible for plant uptake (Guerinot and Yi 1994). Additionally, free Fe is redox-active, which generates reactive oxygen species inside the plant cell. Hence, plants tightly regulate Fe homeostasis to sustain diverse metabolic processes and avoid damage by oxidative stress.

Plants have developed two strategies to obtain Fe (Grillet and Schmidt 2019). Graminaceous plants take up Fe after secretion of Fe^3+^ binding phytosiderophores and subsequent uptake of the Fe^3+^-phytosiderophore complex via an oligopeptide transporter (strategy II) (Grillet and Schmidt 2019). Dicots and non-graminaceous monocot plants employ a reduction-based Fe acquisition mechanism (strategy I), in which Fe^3+^ is first reduced by the Fe^3+^-chelate ferric reductase oxidase 2 (FRO2). The reduced Fe^2+^ is then transported across the plasma membrane by the ZIP family transporter iron regulated transporter 1 (IRT1) (Grillet and Schmidt 2019). Plants modulate the genes’ expression in Fe uptake and transport to maintain Fe homeostasis. Diverse TFs participate in the transcriptional regulation of Fe transport and homeostasis, including the *FER-like iron deficiency-induced transcription factor* (*FIT*) (Colangelo and Guerinot 2004). Under Fe-limiting conditions, four basic helix-loop-helix (bHLH) subgroup IVc TFs (bhLH34, bHLH104, bHLH105/ILR3-IAA-leucine resistant3 and bHLH115) interact with bHLH21 (a bHLH IVb subgroup member) to activate the expression of *FIT* (Zhang et al. 2015, Li et al. 2016, Liang et al. 2017). Then, FIT interacts with four bHLH subgroup Ib (bHLH38, bHLH39, bHLH100 and bHLH101) TFs to form heterodimers that modulate the expression of Fe uptake genes, including *IRT1* and *FRO2*, and other Fe deficiency–responsive genes (**Fig. 1B**) (Yuan et al. 2008, Wang et al. 2013).

Once plants satisfy their Fe needs, they tightly regulate their responses to Fe deficiency to maintain Fe homeostasis. Plants sense Fe levels through hemerythrin motif-containing RING and zinc-finger (HRZ) proteins in monocots (i.e. rice) and in some way by the E3 ligase BRUTUS (BTS) in dicots (e.g. *A. thaliana*) (Kobayashi et al. 2013, Selote et al. 2015). BTS or HRZ proteins interact with bHLH IVc TFs to promote their degradation in the proteasome, further inhibiting FIT’s transcriptional activation (Kobayashi et al. 2013, Selote et al. 2015). To secure the complete shutdown of the Fe deficiency plant responses, POPEYE, another bHLH TF, is accumulated and binds to the promoter region of *bHLH38, bHLH39, bHLH100* and *bHLH101* TFs, which repress their expression, thereby inhibiting the expression of Fe deficiency–responsive genes (Pu et al. 2023). In contrast, when the cell’s Fe levels are low, plants transcribe and accumulate the small peptides IRONMAN (IMA) (Grillet et al. 2018). IMA peptides interact with BTS, allowing bHLH IVc TFs to accumulate and promote the expression of *FIT*, which, in turn, activates the plant responses to Fe deficiency (Li et al. 2021).

Significant progress in understanding the genetic regulation of uptake, transport and homeostasis of Pi and Fe paves the way for improving the efficient use of these nutrients in crop plants, including legumes. Enhancing the efficient use of Pi and Fe in legumes can have a beneficial impact on symbiotic nitrogen fixation, which can reduce our dependence on inorganic fertilizers.

## Pi and Fe Homeostasis under Symbiotic Conditions with Rhizobia

Apart from correctly perceiving and decoding the symbiotic signal, the success of the root nodule symbiosis relies on the efficient nutrient exchange between both partners. When rhizobia stop delivery of fixed nitrogen, the host plant terminates the symbiosis. Similarly, if the host plant does not provide carbon sources or mineral nutrients (e.g. Pi and Fe), the endosymbiont dies and nitrogen fixation is interrupted (Hernández et al. 2009, Udvardi and Poole 2013, Brear et al. 2020). Root nodules require more Fe and Pi than other plant organs do. Root nodules contain a large part of the plant Pi (∼20%) and Fe (∼25-30%) content (González-Guerrero et al. 2014, Sulieman and Tran 2015). Because of the root nodules’ high demand for Pi and Fe, legumes tightly regulate these mineral nutrients’ transport, translocation and homeostasis to sustain nitrogen fixation and plant growth and development.

Legumes have adapted their pre-existing Pi and Fe homeostasis systems to accommodate nodule development and function. The plant genetic machinery to cope with Pi or Fe deficiency operates in the root nodules under optimal nutritional conditions to sustain their high demand for these nutrients (**Fig. 1**). For instance, PHR is active in both rhizobia-infected and non-infected nodule cells of soybean plants growing under Pi-sufficient conditions (Lu et al. 2020). PHR, in turn, activates the expression of PHT1 transporters, PHO1 translocators and other components of the PSR system to secure sufficient Pi in the nodule and sustain nitrogen fixation (**Fig. 1A**) (Lu et al. 2020). Activating the PHR-PHT1-PHO1 modules is crucial to secure Pi for nodule initiation, development and functioning (Lu et al. 2020). Nodules also accumulate PHO2 to mediate the PHT1 and PHO1 turnover, sustaining and coordinating their Pi homeostasis with the whole-plant Pi homeostasis (Huertas et al. 2023).

Regulation of the Pi homeostasis is fundamental to avoid defects in host legume growth and nodule development. Indeed, although overexpression of *PHT1* enhances Pi accumulation in nodules and increases nodule size and nitrogenase activity, this excess of Pi decreases the nodule number in soybeans (Lu et al. 2020). Moreover, overexpression of *PHR1* reduces the number of nodules, whereas a low level of *PHR1* increases nodules regardless of the Pi conditions (Lu et al. 2020). All these data indicate that regulation of Pi homeostasis in the root nodule symbiosis is crucial for optimal nodule development and functioning. Moreover, these data also suggest that fine regulation in the spatiotemporal activity of PHR is fundamental to modulating the activation of the nodule development program.

Legumes use the Fe deficiency genetic machinery to ensure sufficient Fe to nodules under adequate conditions of this micronutrient. For instance, *IMA1* and *IMA2* are upregulated during the root nodule symbiosis in *L. japonicus* (Ito et al. 2024). NIN regulates the transcriptional activation of *IMA1* and *IMA2* under sufficient Fe conditions (**Fig. 1B**). Overexpression of *IMA1* and *IMA2* increases the expression of Fe deficiency–responsive genes, including Fe transporters (i.e. *IRT1*), Fe translocator (i.e. *nicotianamine synthase 2*), the Fe reductase *FRO2* and the TF *FIT* (Ito et al. 2024). Moreover, these *IMA1/2*-overexpressing plants overaccumulate Fe in the nodules and other plant organs, whereas knock-out *ima1/2* plants showed the opposite phenotype and formed 50% more nodules (Ito et al. 2024). All these data indicate that the genetic machinery to cope with Fe deficiency operates in nodules to secure this vital mineral element for symbiotic nitrogen fixation. Furthermore, these data also inform that optimal Fe concentration might be fundamental to activating the nodule development program.

Notably, the role of IMA peptides in regulating Fe homeostasis in the root nodule symbiosis has been reported in *L. japonicus*, which forms determinate nodules (Ito et al. 2024). It is essential to experimentally test whether this mechanism operates in legumes belonging to the inverted repeat-lacking repeat clade (IRLC), which form indeterminate nodules. This testing is crucial as it will to help fill the knowledge gap on the role of IMA peptides in legumes from the IRLC. Despite this gap, significant progress has been made in understanding how these legumes control Fe homeostasis and promote Fe transport to nodules (Sankari et al. 2022). For instance, *M. truncatula*, a member of the IRLC, accumulates the nodule cysteine-rich (NCR) peptide 247 to promote Fe transport to the nodule (Sankari et al. 2022). NCR247 sequesters heme groups, which triggers a Fe-deficient condition in the rhizobial bacteroid even when the host plant grows under Fe-sufficient conditions (Sankari et al. 2022). This heme sequestration scenario overrides the usual Fe homeostasis machinery of the rhizobial bacteroid that, in turn, promotes Fe translocation to nodule-infected cells (Sankari et al. 2022).

Once the Fe deficiency genetic machinery is active, Fe must be transported from the plant-infected cells to the bacteroid. Diverse transporters participate in the Fe transport to nodules and bacteroids. For instance, *NRAMP1*, a member of the Natural Resistance–Associated Macrophage protein family, is highly expressed in roots and nodules, and its protein is located at the plasma membrane, including the plasma membrane of infected cells in *M. truncatula* (Tejada-Jiménez et al. 2015). NRAMP1 is fundamental for apoplastic Fe uptake by rhizobia-infected cells in *M. truncatula* (Tejada-Jiménez et al. 2015). The *Multidrug and Toxic compound Extrusion 67* (*MATE67*) is also highly expressed in the infection zone of nodules from *M. truncatula*, and its protein is located at vascular bundles and symbiosome membrane (SM) (Kryvoruchko et al. 2018). A study in *M. truncatula* indicates that MtMATE67 participates in the Fe transport to the symbiosome (Kryvoruchko et al. 2018). Apart from these transporters, members of the vacuolar iron transporter–like (VTL) family are also fundamental in transporting Fe to the nodules and bacteroids (Hakoyama et al. 2012, Brear et al. 2020, Walton et al. 2020). For instance, *Stationary Endosymbiont Nodule 1* (*SEN1*) is expressed exclusively in rhizobia-infected nodule cells from *L. japonicus* (Hakoyama et al. 2012). *Ljsen1* mutant plants develop nodules with reduced nitrogenase activity and defects in the bacteroid shape (Hakoyama et al. 2012). *GmVTL1a* is a homolog to *LjSEN1* that is also expressed in nodule-infected cells, the protein located at the SM in soybeans (Brear et al. 2020). GmVTL1a is responsible for Fe transport across the SM to bacteroids and is fundamental to sustaining nitrogen fixation in soybean (Brear et al. 2020). VTL4 and VTL8 participate in the Fe transport to bacteroids at different stages of the nodule development in *M. truncatula* (Walton et al. 2020). VTL4 is essential for iron transport at earlier stages of nodule development, whereas VTL8 is crucial for nodule maturation (Walton et al. 2020). Moreover, VTL8, the closest homolog of LjSEN1, participates in delivering Fe to bacteroids in *M. truncatula* (Walton et al. 2020). Ferroportin 2 (FPN2) also plays a fundamental role in Fe delivery to nitrogen-fixing bacteroids in *M. truncatula* nodules (Escudero et al. 2020). MtFPN2 is localized in the intracellular membrane of nodule vascular cells, including endodermis, pericycle and vascular parenchyma (Escudero et al. 2020). Moreover, MtFPN2 is also localized in the SMs within the early fixation zone (Escudero et al. 2020). *fpn2 M. truncatula* mutant plants display an altered Fe distribution in nodules, which reduces the nitrogenase activity and biomass production (Escudero et al. 2020). The participation of diverse Fe transporters indicates the relevance of securing Fe for optimal nitrogen fixation activity in nodules from diverse legumes.

## Genetic Effects of Pi and Fe Availability in the Establishment of the Root Nodule Symbiosis

As stated in the previous section, Pi and Fe are critical nutrients for nodule functioning. Experimental evidence suggests that the availability of Pi and Fe modulates the establishment of root nodule symbiosis. This hypothesis is supported by the fact that the availability of these nutrients affects nodule formation in diverse legumes (Hernández et al. 2009, Slatni et al. 2011, Cabeza et al. 2014, Isidra-Arellano et al. 2020). Fe deficiency can affect either nodule initiation or later development, but the effect of this abiotic stress on the root nodule symbiosis varies between legume species. For instance, *Lupinus angustifolius* growing under Fe-deficient conditions form fewer nodules, indicating a negative effect on nodule initiation (Tang et al. 1990). In contrast, Fe deficiency does not affect nodule initiation in peanut, *Phaseolus vulgaris* and soybean plants but does affect later nodule development (O’Hara et al. 1988, Slatni et al. 2011). Despite these observations, the genetic mechanisms underlying the Fe effect on establishing the root nodule symbiosis are mainly unknown. It is unknown whether Fe and Pi availability affects the perception and decoding of the rhizobial signal.

It is unclear whether Fe availability activates signaling pathways (e.g. AON pathway) to regulate nodule formation. However, experimental evidence shows the relevance of Fe levels in controlling nodule formation. For instance, *ima1/ima2* and *vtl14* mutant plants, which accumulate less Fe, form 50% more nodules than wild-type plants (Walton et al. 2020, Ito et al. 2024). The increase in the number of nodules was reverted when Fe was applied to *ima1/ima2* mutant plants (Ito et al. 2024). Additionally, the overexpression of *IMA1/2* reduced the number of nodules *of har1* mutant plants, which cannot perceive the CLE-RS1 and CLE-RS2 peptides and trigger the AON pathway (Ito et al. 2024). Intriguingly, adding 1 mM Fe partially reverted the hyper-nodulation phenotype of *har1* mutant plants (Ito et al. 2024). These data suggest that the fine regulation of Fe transport and homeostasis might be fundamental to properly activating the nodule development program. Moreover, Fe levels, through the action of IMA peptides, might control the nodule formation by modulation of the AON pathway.

Reduction in the number of nodules is a hallmark in diverse legumes growing under Pi-deficient conditions (Hernández et al. 2009, Cabeza et al. 2014, Isidra-Arellano et al. 2020). Studies in diverse legumes have revealed some genetic mechanisms controlling the root nodule symbiosis under Pi-deficient conditions. For instance, low Pi levels affect the expression of genes participating in flavonoid biosynthesis, compounds that are fundamental to initiating the root nodule symbiosis (Cabeza et al. 2014). Pi deficiency also reduces the expression of critical symbiotic genes, including *NIN*, in *P. vulgaris* plants (Isidra-Arellano et al. 2018). Hence, Pi availability might have a negative impact recognizing rhizobia by the root and likely in decoding the rhizobial signal.

Studies in soybean and *P. vulgaris* suggest that the TF PHR1 might be fundamental in regulating the nodule development program (Isidra-Arellano et al. 2020). Soybean plants overexpressing *PHR1* develop fewer nodules, whereas plants with low *PHR1* levels develop more nodules regardless of the plant host Pi status (Lu et al. 2020). Interestingly, critical components of the AON pathway (i.e. *RIC1, NIN* and *TML*) contain at least one P1BS *cis*-regulatory element, which PHR1 binds, suggesting that PHR1 might modulate their expression according to the plant host Pi status (**Fig. 1A**) (Isidra-Arellano et al. 2020). Additionally, *nark* (ortholog of *HAR1/SUNN* receptors) mutant plants display no nodule number reduction when growing under Pi-deficient conditions (Isidra-Arellano et al. 2020). All these data indicate that plant host Pi status is fundamental for modulating the activation of the AON pathway to control nodule formation under this nutritional condition. These data also suggest that PHR might be critical in activating the AON pathway according to the plant host Pi status. Finally, PHR can be a crucial genetic component of the root nodule symbiosis because of its positive role in securing enough Pi to the nodule and its negative role in controlling the nodule formation. However, further experimentation is needed to understand the potential dual role of PHR in the root nodule symbiosis.

## The Potential Genetic Interplay between Nitrogen-Fe-Pi in the Root Nodule Symbiosis

The nitrogen, Pi and Fe uptake and homeostasis are intimately interconnected (Medici et al. 2019). A deficiency of one of these nutrients can lead to excess accumulation of the other nutrients and vice versa. For instance, high levels of free Fe can rapidly interact with Pi, making Pi inaccessible for the plant, hence triggering Pi deficiency (Vance et al. 2003). Similarly, high Pi levels can trigger a Fe-deficient condition in diverse plant species (Vance et al. 2003). Therefore, plants must tightly coordinate the uptake and homeostasis of nitrogen, Pi and Fe to avoid defects in growth and development.

**Fig. 2:**
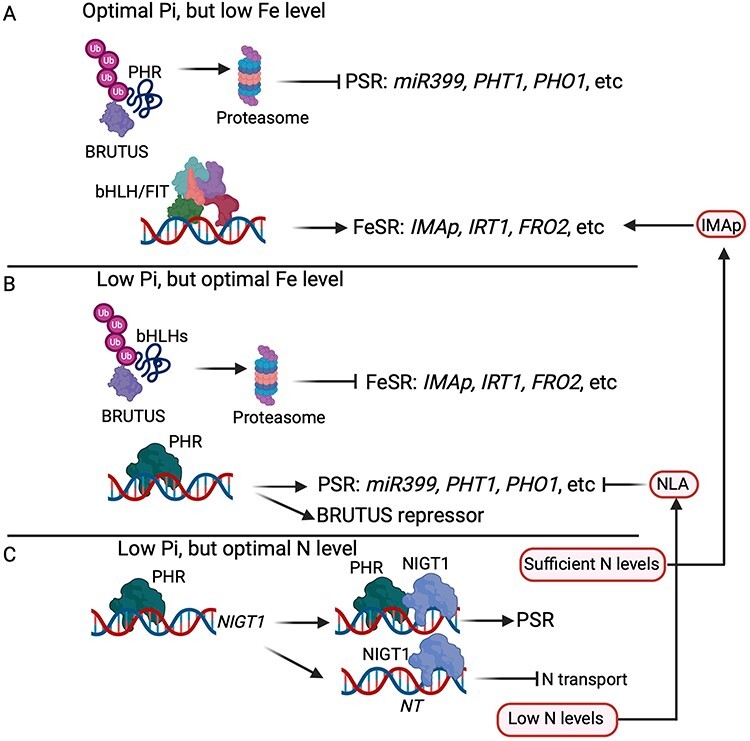
Genetic interplay in regulating the Pi, Fe and nitrogen uptake and homeostasis under non-symbiotic conditions. Under optimal Pi conditions, Fe forms chemical complexes with Pi, making it inaccessible, which triggers a low-Fe condition in the plant cell. Under this scenario, the ubiquitin E3 ligase BTS interacts with PHR, promoting its degradation in the proteasome, further inhibiting the PSR system, including the miRNA *miR399, phosphate high-affinity transporters 1* (*PHT1*) and *Pi translocator* (*PHO1*) and other genes. Simultaneously, bHLH and FIT interact and activate the expression of the FeSR system, which includes *IMAp*, *iron transporter 1* (*IRT1*), *FRO2* and other genes required to transport Fe and control its homeostasis. (B) Once the plant satisfies its Fe need, BTS interacts with bHLH TFs, promoting their degradation at the proteasome, further inhibiting the FeSR system. Then, active PHR activates the PSR system to provide Pi to the plant. (C) Pi deficiency inhibits the nitrogen (N) transport. Under low-Pi conditions, PHR activates the expression of *NIGT1*, a repressor of nitrogen transporter expression. PHR and NIGT1 activate the PSR system to fulfill the plant’s Pi requirements. Once a plant fulfills its Pi needs, nitrogen levels often are lower. This condition promotes the activation of the ubiquitin E3 ligase NLA1, which contributes to shutting down the Pi transport. Interestingly, when the plant satisfies its nitrogen need, IMAp is produced to activate the FeSR system, promoting Fe transport and homeostasis. Images were created using BioRender (https://biorender.com).

Significant progress has been made in understanding the genetic mechanisms modulating their signaling and homeostasis (Medici et al. 2019). Interestingly, PHR is a crucial component that integrates the nitrogen, Pi and Fe levels and modulates their transport and homeostasis according to the plant’s needs in rice and *A. thaliana* (**Fig. 2**) (Ueda et al. 2020, Guo et al. 2022). For instance, under Fe-deficient conditions, the E3 ligases HRZ/BTS, Fe sensors in plants and ubiquitinate PHR to promote its degradation at the proteasome in rice and *A. thaliana* (**Fig. 2A**) (Kobayashi et al. 2013, Selote et al. 2015, Guo et al. 2022). The PHR degradation further inhibits the activation of the PSR system, including Pi transporters, which, in turn, allows rice plants to activate the responses to Fe deficiency and fulfill their Fe needs (**Fig. 2A**) (Guo et al. 2022). In line with this, *hrz/brutus* mutant plants accumulate more PHR protein and Pi but less Fe in rice (Guo et al. 2022). Interestingly, when a plant satisfies its Pi needs, the active PHR indirectly represses the expression of *HRZ/BTS*, allowing rice plants to activate the molecular responses to transport Fe and sustain its homeostasis (**Fig. 2B**) (Guo et al. 2022). All these observations confirm that a component of the Fe homeostasis also participates in controlling Pi transport and homeostasis and that PHR is fundamental in coordinating the uptake and homeostasis of Pi and Fe. However, the participation of these genetic components in controlling the coordinated Fe and Pi homeostasis must be investigated in legumes while symbiotically interacting with rhizobia.

Pi deficiency in diverse plant species reduces nitrogen uptake, indicating that these two macronutrients’ uptake and homeostasis are tightly regulated (Ruffy et al. 1990, Medici et al. 2019). In recent years, significant advances have been made in understanding the molecular mechanisms underlying the coordination of nitrogen and Pi uptake and homeostasis in *A. thaliana* (Maeda et al. 2018, Medici et al. 2019). This genetic regulation involves the nitrate-inducible GARP-type transcriptional repressor 1 (NIGT1) and PHR (Maeda et al. 2018). Interestingly, the expression of *NIGT1* is also regulated by low-Pi conditions in diverse plant species, suggesting its participation in regulating nitrogen and Pi uptake and homeostasis (Medici et al. 2015). NIGT1 is a negative regulator of *nitrate transporter 2.1* (*NRT2.1*), which encodes a major high-affinity nitrate transporter indispensable for diverse plant species to uptake nitrate under nitrogen-deficient conditions (Orsel et al. 2004). Under Pi-deficient conditions, PHR activates the expression of *NIGT1*, which, in turn, represses the expression of *NRT2.1* (**Fig. 2C**) (Maeda et al. 2018). Interestingly, NIGT1 and PHR activate the PSR system, allowing the plant to satisfy its Pi needs (Nagarajan et al. 2016). Once the plant fulfills its Pi needs, the ubiquitin E3 ligase NLA accumulates and contributes to the degradation of PHT1 transporters and activates the nitrogen uptake (**Fig. 2C**) (Kant et al. 2011). This accumulated knowledge confirms the interconnection of nitrogen and Pi signaling pathways to control the uptake and homeostasis of these macronutrients and avoid defects in plant growth and development. However, the participation of these genetic components in regulating the coordinated homeostasis between nitrogen and Pi is yet to be investigated in legumes symbiotically interacting with rhizobia. Hence, it is imperative to determine whether this genetic machinery is required for a successful root nodule symbiosis.

NRT2.1 is also a fundamental regulator of nodule formation in *L. japonicus* and *M. truncatula* (Misawa et al. 2022, Luo et al. 2023). MtCEP1 peptides systematically induce the expression of *NRT2.1* under low-nitrogen conditions, promoting nodule formation in *M. truncatula* (Luo et al. 2023). However, under sufficient nitrogen conditions, NLP1 induces the expression of *NRT2.1*, repressing the nodule formation in *M. truncatula* and *L. japonicus* (Misawa et al. 2022, Luo et al. 2023). Moreover, the CEP1 peptide promotes nitrogen uptake through NRT2.1 in *A. thaliana* and *M. truncatula* (Tabata et al. 2014, Luo et al. 2022). Applying synthetic CEP1 peptide promotes the Pi uptake through PHT1 transporters in both model plants (Roy et al. 2022). The fact that the Pi deficiency inhibits the expression of *NRT2.1* through NIGT1 and that NRT2.1 is fundamental to promoting nodule formation adds evidence supporting the notion that coordinated homeostasis between nitrate and Pi is instrumental in promoting nodule formation in legumes. However, further research is needed to demonstrate that Pi deficiency reduces the expression of *NRT2.1* in legumes while symbiotically interacting with rhizobia. Furthermore, the fact that CEP1 is instrumental in promoting nodule formation and that this peptide also promotes Pi uptake in *M. truncatula* suggests that optimal Pi levels might be required to activate the nodule development program. Indeed, Pi deficiency upregulates the expression of *CEP1*, and its overexpression promotes the symbiosis with arbuscular mycorrhizal (AM) fungi in *M. truncatula* (Pedinotti et al. 2024). Additionally, *cra2 M. truncatula* mutant plants show a reduction in symbiosis with AM fungi (Pedinotti et al. 2024). As *CEP1* is induced by low-nitrogen and low-Pi conditions, this peptide and CRA2 might coordinate the root competency for rhizobia or AM fungi depending on the nitrogen and Pi availability combination. According to this hypothesis, CEP1 and CRA2 might promote symbiosis with rhizobia under a low-nitrogen and optimal Pi scenario. However, the scarcity of both nutrients might facilitate the interaction with AM fungi.

The expression of genes encoding the IMA peptides is also regulated by nitrogen in *L. japonicus* and *A. thaliana* (**Fig. 2C**) (Ito et al. 2024). Knock-out *ima* plants accumulate more nitrogen but less Fe than wild-type plants. Adding Fe to *ima* plants reverts this phenotype, indicating that nitrogen uptake reduces Fe uptake and homeostasis (Ito et al. 2024). In line with this, high-nitrogen conditions upregulate the expression of *IRT1* and *FRO2*, confirming that high-nitrogen conditions trigger a Fe-deficient response (Ito et al. 2024).

Maintaining the homeostasis of nitrogen, Pi and Fe is fundamental to avoid a toxic or deficient condition of one of these nutrients that affect plant growth and development. Most of the described knowledge in this section is under a non-symbiotic scenario. Hence, how do legumes regulate the uptake and homeostasis of these three critical nutrients while symbiotically fixing nitrogen? Therefore, it is imperative to investigate whether the genetic interplay between nitrogen, Pi and Fe operates under symbiotic conditions with rhizobia or whether other genetic components, including peptides and miRNAs, operate under this condition. We know that *ima1/2* mutant plants hyperaccumulate nitrogen, which reduces the number of nodules in *L. japonicus* (Ito et al. 2024). Another example of the relevance of the nutritional interplay in the root nodule symbiosis is the regulation of the *miR399* expression, a Pi-deficient responsive miRNA that promotes Pi uptake in diverse plants, including legumes (Bari et al. 2006, Argirò et al. 2024). *MiR399* upregulation is promoted when the plant’s nitrate levels are optimal, whereas low nitrogen levels reduce its expression in *M. truncatula* (Argirò et al. 2024). Moreover, *cra2* mutant plants or transgenic roots overexpressing *CEP1* display reduced levels of *miR399* (Argirò et al. 2024). Interestingly, the overexpression of *miR399* significantly reduces the number of nodules in *M. truncatula* (Argirò et al. 2024). These recent data indicate that the CRA2/CEP1 pathway modulates the *miR399* expression according to the nitrogen availability, which is fundamental to promote or restrict the nodule formation. Furthermore, all these data add more evidence supporting the notion that regulating the uptake and homeostasis of these nutrients is essential to establishing the symbiosis with rhizobia and sustaining the nitrogen fixation process. Thus, the next movement in this research field is to understand how legumes genetically regulate the homeostasis of nitrogen, Pi and Fe to maintain a successful symbiosis with rhizobia.

## Perspectives and Conclusions

Over the past 20 years, significant progress has been made in understanding the genetic mechanisms underlying the establishment of root nodule symbiosis and nodule functioning. This knowledge has been instrumental in pursuing the goal of transferring the nitrogen fixation trait to cereals. However, root nodule symbiosis is negatively affected by the availability of Pi and Fe, a nutritional condition common in arable soils around the globe. The coordinated homeostasis between nitrogen, Fe and Pi activates the symbiotic genetic program. Recent evidence indicates that Pi and Fe plant host levels have a significant impact on the activation of the genetic programs required for nodule formation. The AON pathway likely modulates the nodule formation program according to the Pi and Fe levels. Intriguingly, PHR is a convergence point that coordinates and integrates inputs and outputs from Pi, Fe and nitrogen plants’ host levels and modulates the host plant response to rhizobia accordingly. The need for a thorough investigation into how legumes coordinate the homeostasis of these three nutrients and secure a successful nitrogen-fixing symbiosis is emphasized. Moreover, it is crucial to research how legumes coordinate the potential dual role of PHR in securing Pi to the nodule and restricting the interaction with rhizobia when Pi is unavailable. Although recent evidence indicates that Fe might activate the AON pathway, it is necessary to understand how Fe levels modulate the symbiotic genetic programs and whether this response is legume species–specific. Finally, it is imperative to determine whether the availability of Fe and Pi impacts decoding the rhizobial signal or whether the availability of these nutrients impacts nodule formation only. This knowledge about the genetic effect of Pi and Fe availability is crucial to improving nitrogen fixation in legumes and, at some point, in cereals.

## Data Availability

The paper provides source data for **Figs. 1** and **2**. This study did not generate or analyze new datasets.
